# Accuracy between virtual surgical planning and actual outcomes in orthognathic surgery by iterative closest point algorithm and color maps: A retrospective cohort study

**DOI:** 10.4317/medoral.22724

**Published:** 2019-03

**Authors:** Daniel-Amaral-Alves Marlière, Maurício-Silva Demétrio, Alan-Robert-Moreira Schmitt, Caio-Bellini Lovisi, Luciana Asprino, Henrique-Duque-de Miranda Chaves-Netto

**Affiliations:** 1 DDS, MSc, PhD Student. Division of Oral and Maxillofacial Surgery, Piracicaba Dental School, State University of Campinas, São Paulo, Brazil; 2 DDS. Oral and Maxillofacial Surgeon and Oral and Maxillofacial Radiologist, Private Office, Maranhão, Brazil; 3 DDS, MSc. Oral and Maxillofacial Surgeon, Private Office, Minas Gerais, Brazil; 4 DDS, PhD. Division of Oral and Maxillofacial Surgery, Piracicaba Dental School, State University of Campinas, São Paulo, Brazil; 5 DDS, PhD. Department of Clinical Dentistry, Juiz de Fora Dental School, Federal University of Juiz de Fora, Minas Gerais, Brazil

## Abstract

**Background:**

To evaluate the accuracy between actual outcomes and virtual surgical planning (VSP) in orthognathic surgery regarding the use of three-dimensional (3D) surface models for registration using iterative closest point (ICP) algorithm and generated color maps.

**Material and Methods:**

Construction of planning and postoperative 3D models in STL files format (M0 and M1, respectively) from CBCT of 25 subjects who had been submitted to bimaxillary orthognathic surgery was performed. M0 and M1 were sent to Geomagic software in semi-automatic alignment surface mesh order of M0 and M1 for registration using ICP algorithm to calculate mean deviation (MD, MD+, MD-, SD) and root mean square (RMS – 3D Error). Color maps were generated to assess qualitative congruence between M0 and M1. From deviation analysis, 3D Error was defined as accuracy measurement. To assess the reproducibility, the workflow was performed by two evaluators multiple times. t-tests were used to assess whether all means of MD, MD+, MD-, SD and 3D Error values would be ≤ - 2 mm and ≥ 2 mm.

**Results:**

High intra and inter evaluators correlation were found, supporting the reproducibility of the workflow. t-tests proved that all MDs and 3D Error values were > - 2 mm and < 2 mm.

**Conclusions:**

3D error mean was within the standards of clinical success lower than 2 mm. ICP algorithm provided a reproducible method of alignment between 3D models and generated color maps to evaluate 3D congruence but did not answer all methodological parameters regarding the assessment of accuracy in orthognathic surgery.

** Key words:**Accuracy, Cone-Beam computed tomography, CAD-CAM, Orthognathic surgery, three-dimensional imaging.

## Introduction

When the focus is a correction of dentofacial deformity, current advances in virtual surgical planning (VSP) of orthognathic surgery have been valuable for diagnosis, treatment planning and outcome evaluation (1,2). The VSP is a combination fan beam or cone-beam computed tomography (CBCT) with software tools to allow three-dimensional (3D) treatment planning in orthognathic surgery which provides to surgeons an opportunity to perform 3D virtual osteotomy, 3D soft tissue simulation, 3D-based surgical splint manufacturing and finally 3D superimposition to evaluate between predictable planning and favorable surgical outcome (3).

3D methods for evaluating the accuracy of the postoperative outcome regarding the VSP had been proposed in previous studies (4-6). The most commonly used method was linear and angular measurements based on cephalometric landmarks to quantify differences between the VSP and postoperative outcome. There was an inherent shortcoming of the landmark-based analysis because this method could be an incomplete evaluation, needed to identify the same landmarks multiple times and did not eliminate human error (3,6).

Hernández-Alfaro and Guijarro-Martinez scanned the intra-operative position of dentitions in the intermediate occlusal guide using an intra-oral scanner and compared these surfaces to the planned objects. The 3D surfaces were submitted to the Iterative Closest Point (ICP) algorithm registration that provided a color map diagram, mean and standard deviations of the difference in distances between the surface’s superimpositions (7). This method of assessing changes in 3D surfaces involves measuring the point-to-point distance of one mesh (VSP - 3D model reference) to the second mesh (Postoperative - 3D model test) and generating a color distance map (8). Distance measurements are automatically performed by the software and represent differences between the superimposed surfaces, then, these distances are depicted in a graphical format as a color representation. Histograms show positive colors which depict regions that are in front of the reference surface indicating outward movements, and, negative colors, regions that are behind the reference surface demonstrating backward movements (9). As positive and negative values could cancel each other during the calculation of the signed average, it cannot be considered as an accurate representation of the actual error. Instead of computing means deviation, the root mean square (RMS) will be able to be useful even if distance measurements can be both positive and negative, as the distances are squared before being averaged (10).

The ICP algorithm and color-coded maps had been applied to quantify accuracy of 3D prediction of soft tissue changes following orthognathic surgery (11); to assess accuracy and reproducibility of the voxel-based superimposition method of CBCT through RMS distance measurements between 3D surface models (12,13); to evaluate the accuracy of superimposition between CBCT and 3D photographic data (10); to overlap 3D face model acquired via different optical facial scanners of dentofacial deformities patients and calculate RMS as a 3D measurement indicator by reverse engineering software - Geomagic Studio (14).

Recently, the accuracy between VSP and surgical outcomes has been highly investigated by different assessment methods that were not compared between themselves and not recognized at the same level (6). Even though two systematic reviews were published commenting that the success of results in orthognathic surgery relies on the accurate transfer of surgical planning, deviations between VSP and actual results lower than 2 mm were not considered clinically significant (4,5).

Therefore, the aim of this study was to evaluate the accuracy of actual outcomes in orthognathic surgery regarding VSP using 3D surface models to registration ICP algorithm and generation color maps by reverse engineering software.

## Material and Methods

**Figure 1:**
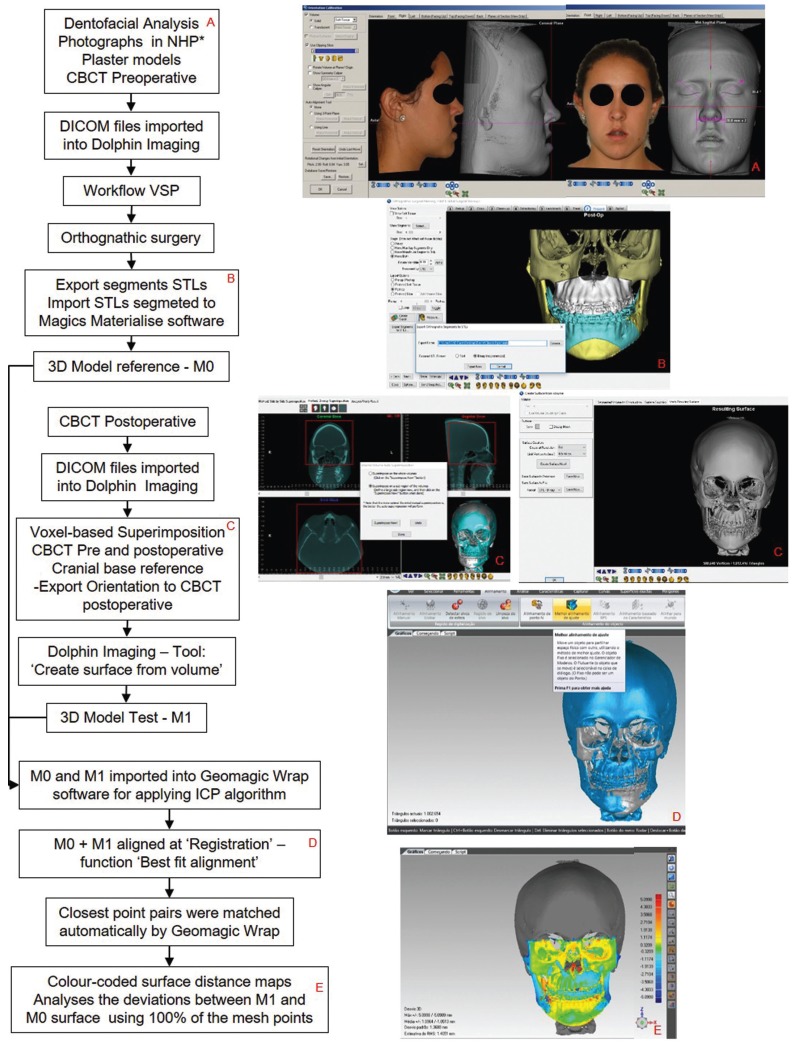
Scheme showed (A - B) workflow of VSP until generating M0; (C) voxel-based superimposition until generating M1; (D – E) application of ICP algorithm between M0 and M1 and color-coded distance map generation. *Natural Head Position.

This retrospective cohort study was approved by the Research Ethics Committee of the Federal University of Juiz de Fora (CAAE 695.98017.2.0000.5133). The sample comprised the CBCT files obtained from twenty-five adult subjects who were submitted to bimaxillary orthognathic surgery between October 2015 and April 2017. The CBCT scans were taken pre and immediately post-orthognathic surgery. The images were acquired with the i-CAT scanner (Imaging Sciences International LLC, Hatfield, PA, USA) with a 22x16 cm FOV, scanning time of 17 s, set at 120 kVp, 5 mA, isotropic 0,4 mm voxel size and grey levels of 14 bits. The DICOM (Digital Imaging and Communication in Medicine) files were imported into Dolphin Imaging 11.7 Premium software (Dolphin Imaging and Management solutions, Chatsworth, CA, USA). The treatment planning protocol and surgical procedure were conducted following the same workflow for all subjects and operated by the same team of surgeons (Fig. 1).

Inclusion criteria for the study were as follow (a) availability of pre and postoperative CBCT data imported into Dolphin Imaging software; (b) availability of VSP; (c) bimaxillary orthognathic surgery through Le Fort I osteotomy and bilateral sagittal split osteotomy. Exclusion criteria were: Patient’s CBCT data with (a) craniofacial syndromic abnormalities, cleft palate, degenerative condylar disease, sequels of facial trauma, and previous history of Le Fort I osteotomy or bilateral sagittal split osteotomy (b) additional operation at the time such as multi-segment Le Fort I osteotomy, chin osteotomies, mandibular subapical osteotomy and transoral vertical ramus osteotomy. This study was limited to single-segment Le Fort I and bilateral sagittal split osteotomy in order to facilitate the comparative evaluation of the deviations without the influence of other osseous changes and the absence of use of an occlusal guide.

Each subject in the database was characterized by age; gender; malocclusion; type of dentofacial deformity; facial proportion (symmetry or asymmetry); surgical treatment planning (rotation of the maxillomandibular complex in clockwise or counterclockwise direction, and anteroposterior movements); surgical sequence (maxilla first, normal sequence; mandible first, inverted sequence).

-CBCT volume superimposition and 3D image processing 

After workflow of VSP, each preoperative CBCT archive was converted to a surface mesh and saved as segmented files STL format (Standard Tessellation Language). One observer (M.S.D) imported all segmented STL files to Materialise Magics software (Materialise NV, Leuven, Belgium) to match with the Merge Selected Parts tool, then generate reference 3D models that were exported as STL files categorized M0 (Fig. 1).

The postoperative CBCT volumes were superimposed over the preoperative CBCT volumes by the same observer (M.S.D) using Dolphin Imaging. Axial, sagittal, and coronal slice views of the volumes were used to select the cranial base anatomical structures in the CBTC volumes. Next, Dolphin Imaging automated method Superimpose now tool optimally aligned the postoperative CBCT to the preoperative CBCT (Fig. 1). These voxel-based superimposition procedures were used to maintain the same pre and postoperative head position (12-13,15). After the superimposition had been done, one observer (M.S.D) was responsible for exporting the 3D model’s test (categorized M1) as STL files using Dolphin Imaging software with the Create surface from volume tool at full resolution (Fig. 1).

The STL files of reference 3D models (M0) and test (M1) obtained from Dolphin Imaging and Materialise Magics software were imported into Geomagic Wrap 2013 software (3D System, California, USA) to analyze the 3D deviations between M0 and M1.

-3D accuracy evaluation

For each pair of model STL file imported into Geomagic Wrap software, the ‘Registration’ function of the software was used to superimpose datasets in the following steps: M0 as a fixed 3D model and M1 as a floating 3D model were submitted to the Best fit alignment function, calibrated in sample size 5000, tolerance value 0, and selected options: verify symmetry (exhaustive), fine adjustments only, high precision mounting and elimination of automatic diverter. The software used the ICP algorithm to adjust the position of the floating 3D model (M1) automatically and exactly overlapping fixed 3D model (M0). This automatic alignment presented values of registration errors as Mean Error and RMS Error (Fig. 1).

To evaluate the 3D deviations between M0 and M1 the deviation analyses function was applied using ICP algorithm in the same software for 3D comparison and measurements between reference and test surface mesh. This software function was calibrated with maximum and minimum deviation values ± 5 mm, critical angle of 45º, fine resolution, and scale of 14 colors. The ICP algorithm calculated the closest point distance between thousands of surface triangles in the 3D surface models (M0 and M1), providing the color-coded surface distance maps that allowed quantification measurements as Mean Deviation (MD), Mean Deviation positive (MD+), Mean Deviation negative (MD-), Standard Deviation (SD), Root Mean Square (RMS). Color difference images were output to examine the congruency of M0 and M1 qualitatively (Fig. 1).

**Figure 2:**
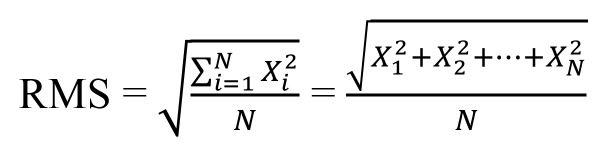
Formula.

All deviations between the closest point pairs on the M1 and M0 were matched and calculated automatically by the algorithm of the software. The value of RMS was calculated using the following formula: (Fig. 2).

If a point P on the M0 had the closest point P’ on the M1, then X is the distance between P and P’, and N is the total number of point pairs on both models. The RMS involved the following steps: 1) all deviations values squared; 2) the squares were added together, and their average was calculated, 3) the square root of the resulting average was estimated (10). The RMS was defined as 3D error which can serve as a measurement indicator of how far deviations between two different datasets vary from zero (14).

These workflows were performed by two evaluators (EVA1 - D.A.A.M and EVA2 - H.D.M.C.N) and repeated after 10 days to check reproducibility. The results were exported to an excel spreadsheet.

-Statistical analysis

Statistical data analysis was performed with R Core Team software, version 3.4.2 (R Foundation for Statistical Computing, Vienna, Austria). Seven variables of the sample characteristics (six categorical and one numerical) were submitted to descriptive statistical analysis. Pearson correlation coefficients (ccP – r) were calculated to assess the intra and inter evaluators (EVA1 and EVA2) agreement regarding the calibration method of the automatic alignment (registration error) and deviation analyses function (MD and 3D Error) between M0 and M1.

The mean and standard deviation (SD) were calculated to Mean Error, RMS Error, MD, MD+, MD-, SD and 3D Error (deviations between M0 and M1). The paired t-test was used to calculate the difference between evaluators, there is adopted the null hypothesis of similarity between above-mentioned means (H0: µ = µ0). To evaluate if success of clinical results in orthognathic surgery were kept on the deviations ≤ 2mm between VSP and actual outcomes, test t was applied to reject or not the null hypothesis established with mean of MD, MD+, SD and 3D Error always equal or greater than 2 mm (H0: µ ≥ 2 mm), thus mean MD and MD- applied the alternative null hypothesis which was always equal or lower than -2 mm (H0: µ ≤ -2 mm). Statistical significance was set at *p* ≤ 0,05.

## Results

From CBCT data archived in Dolphin Imaging, twenty-five subjects were included and the mean age for the sample was 27 years. Thirteen (52%) and Twelve (48%) were male and female, respectively. Most subjects were classified as class III malocclusion (84%). The sample consisted mostly of dentofacial deformity with retrognathia of the upper jaw associated to prognathia of lower jaw (64%), and asymmetric facial proportion (52%). Regarding surgical treatment planning, 44% of the patients were planned by rotation of the maxillomandibular complex in a counterclockwise direction and 72% undergoing mandible first (inverted sequence).  Table 1 showed all frequencies and percentages of each sample characteristic.

**Table 1:**
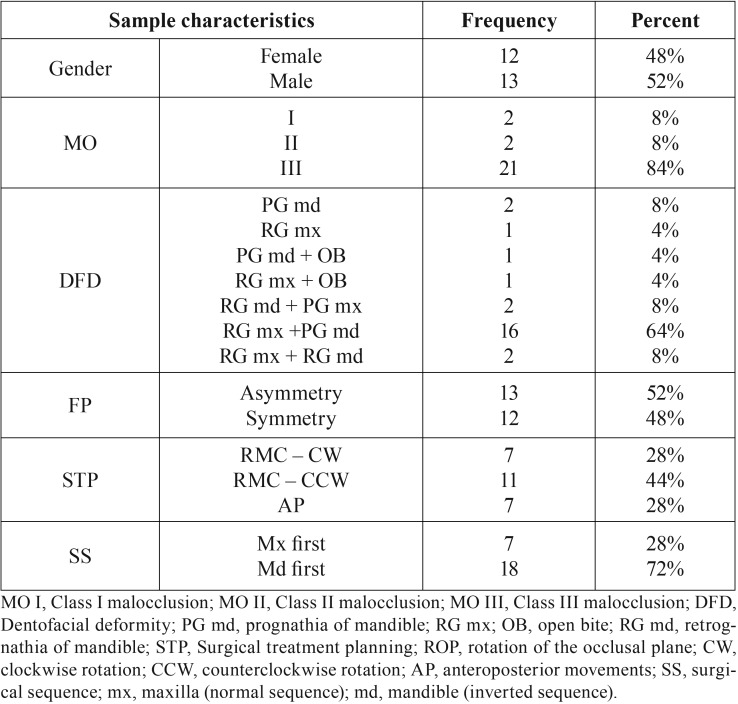
Descriptive statistics distribution of sample characteristics.

**Figure 3:**
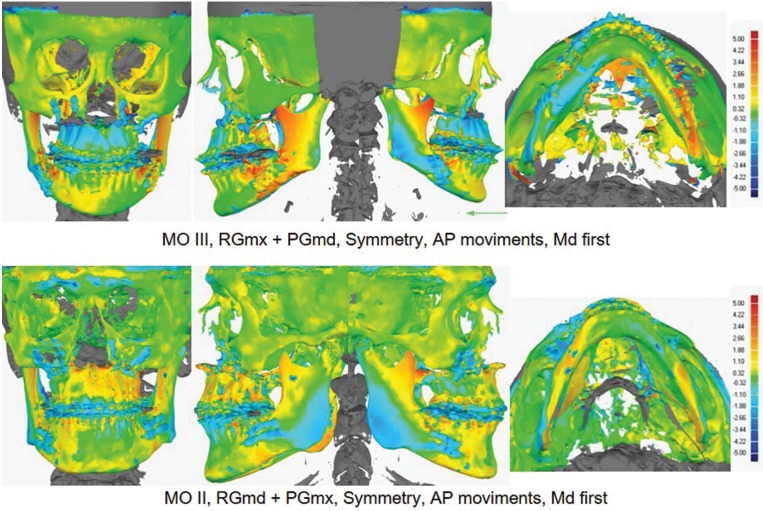
Illustrations showed examples of sample subjects that underwent orthognathic surgery and accuracy evaluation. Color maps overview qualitative deviations between M0 and M1 and histograms ± 5 sign depicts the distance or deviation range. Green color indicated zero deviation, warner (red) colors positive deviations, and colder (blue) colors negative deviations.

Each 3D model test (M1) was superimposed on the 3D model reference (M0), and then, these generated visual displays of magnitude and location of disagreement or congruence between models. The qualitative results could be seen by color maps predominantly green in the regions of the orbit, zygomatic and skull base, as deviations quantified at zero between M0 and M1. Generally, distal segments of maxilla showed range of colors (green, yellow and light blue) and proximal segments of mandible also presented range of colors (blue, green - yellow, and yellow – red, respectively) in the regions of the mandible angle, condyle and coronoid process (Fig. 3).

 Table 2 shows the ccP and Paired t-test results, all the values for ccP were higher than 97% (r ≥ 0,97). The Paired t-test did not reject the null hypothesis of similarity (H0: µ0 = µ) between means of registration errors and deviations comparing the two evaluators, all results were not statistically significant (*p* ≥ 0,05). These results confirmed the excellent reproducibility of the intra-evaluator and inter-evaluators method.

**Table 2:**
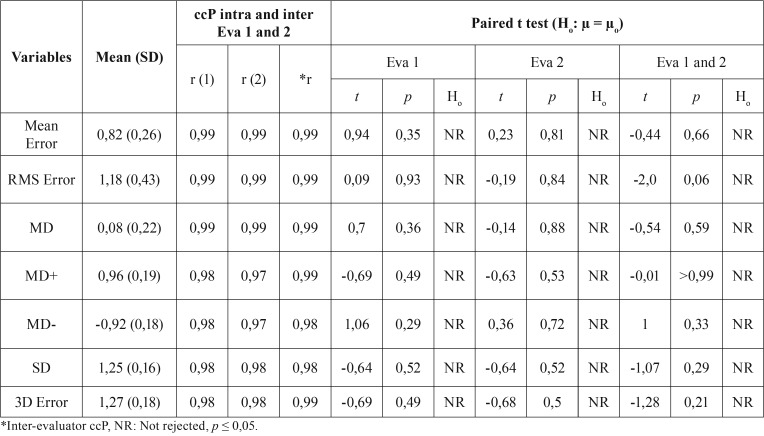
Pearson correlation coefficients (ccP – r) and Paired t-test intra-evaluator and inter-evaluators to test the reproducibility.

**Figure 4:**
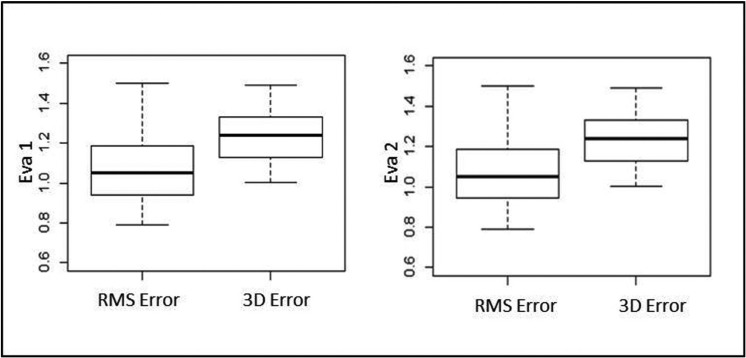
Illustration showed boxplots with values lower than 2 mm and congruence of the graphics, comparing RMS Error and 3D Error between EVA(s).

The boxplots show the congruence of the results between two evaluators to RMS Error and 3D Error (Fig. 4). For EVA 1 and EVA 2 were obtained similar values of means (1,18 mm and 1,27 mm, respectively). To confirm that all mean values respected the clinical success criterion reported (7),  Table 3 presented the null hypothesis that was rejected (*p* < 0,05), and t-test showed all mean values of the seven deviation variables were > - 2 mm and < 2 mm.

**Table 3:**
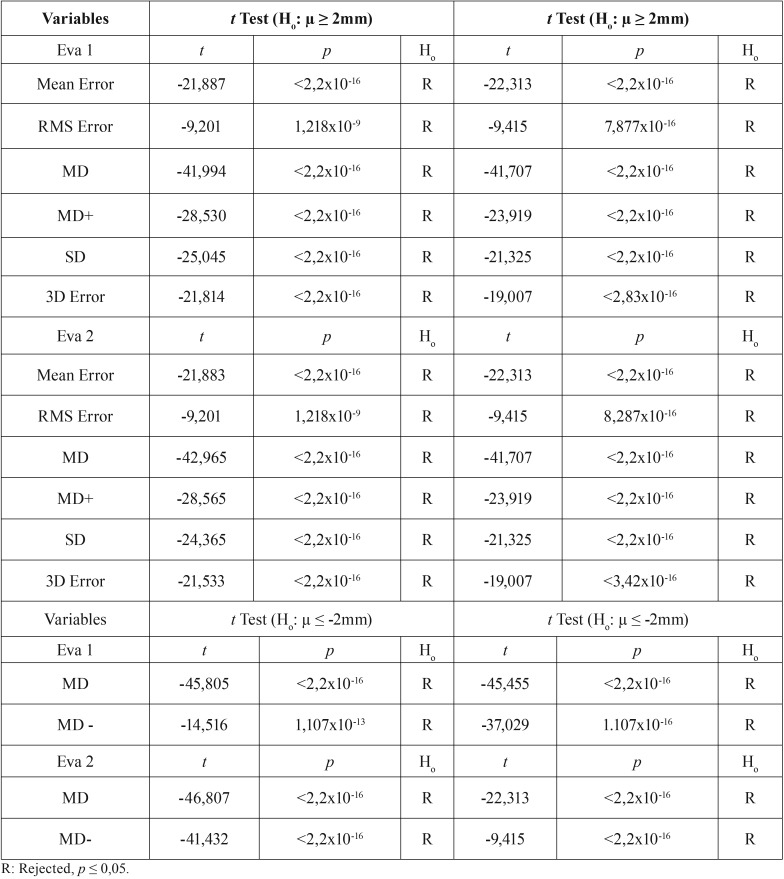
*t*-Test analysis to assess all means of registration error and deviations rejected or not the hypotheses.

## Discussion

When cephalometric tracings were used for treatment planning in orthognathic surgery, the maximum deviation tolerance between planning and outcomes, in both soft and hard tissues, was 2 mm (16). Nowadays, several authors still propose that the success criteria remains of a difference of maximum 2 mm between the VSP and actual outcomes, there have been maximum tolerance levels shown to not be clinically significant (4,17-20). As shown in the results based on the RMS deviations between the surface meshes of 3D Models (M0 and M1), the 3D error was lower than the established 2 mm maximum tolerance level for hard tissues ( Table 2).

The 3D error was used as a measurement indicator for our method, which can indicate the 3D shape congruency of the hard tissues on the M0 and M1. It can express more 3D shape information, being more comprehensive than methods used in previous studies that were based on the calculations of linear and angular differences between cephalometric landmarks (2,18,21) or the computation of intra-class coefficients of reference points and reference angles (7). In aforementioned methods, landmarks or reference points need to be identified multiple times on the virtual 3D models by the observer, but these measurements were based on identifying landmarks or points which was prone to human error ranging from 0,02 to 2,8 mm (22). Therefore, other assessment methods could be applied to overcome observer-dependent landmark identification errors, reducing possible influences on clinical interpretation of the results and eliminate the risk of bias (3).

In this sense, different assessment methods were possible because dentofacial deformities are 3D in nature and so VSP made it easy to evaluate the movements or changes of the underlying skeletal hard tissue (planning in comparison to the actual results) (4,5). Currently, CBCT is the favored method to obtain and visualize images of hard tissues in many ways (viewing the slice data, direct volume rendered 3D and 3D surface model rendering) (8). In this study, 3D surface model rendering method was used which resulted in the production of polygonal meshes (surface of points or triangle vertices with known 3D coordinates), being accepted as the easiest, most clinically useful, and the least computationally intensive method (8).

Marchetti *et al.*, Mazzoni *et al.* and Tucker *et al.* performed previous studies that included 10, 25, and 20 patients, respectively, using similar automatic methods to evaluate accuracy between VSP and postoperative outcome. For theses authors, the methods were done using a surface-to-surface best fit of the two virtual models aligning the base of the skull and measuring the distance between the planned and actual outcome postoperatively. The measurements were presented as the mean distance between the geometric models within 2 mm of the planned outcome (17,20). Mazzoni *et al.* used the intraoperative navigation to improve accuracy and had mean surface differences of less than 2 mm; a mean matching error of 1,09 mm was reported, and less than 2 mm on 86.5% of all surfaces.

In the present study, our method comprised the semi-automatic evaluation in order to minimize human error and facilitate the application by surgeons in their daily practice because the surgeons would still select the parameters settings in Geomagic Wrap software. Widely accepted by surgeons, the reproducibility of our method was calculated by intra and inter-assessor reliability correlation, for which high agreement level was observed ( Table 2). Despite the facility of this correlation form, it does not give an indication of the quality or accuracy of the model used (6).

Color-coded distances are an analytical tool incorporated in most computer-aided design software packages to measure the relative distance or deviation between two 3D surface meshes (23). Previous studies had used the color mapping method to assess 3D hard tissue displacement (8,24). Generally, a green color indicates zero deviation, signaling suitable automatic alignment method between surface meshes of 3D models (M0 and M1). The green, yellow or light blue colors were preponderantly seen at the distal segments of the maxilla and presented a bigger range of colors in the regions of the mandible angle, condyle and coronoid process. Despite the color variability, the bone segments that correlated more closely with occlusal splint presented lower variation and intensity of colors, indicating that the occlusal splints transferred the surgical planning more precisely to the proximal bone segments (Fig. 3). However, there was variability of colors in the distal segment of the maxilla that could be related to the occlusal splints that did not improve vertical control of the maxilla (1,7). The bilateral sagittal split osteotomy can undergo interferences by the medial pterygoid muscle and stylomandibular ligament when the distal segment was setback and slid past the lingual aspect of the proximal segment. For mandibular advancement procedures, the muscle attachment is split with one part attached to the proximal segment and the other part attached to the advanced distal segment, which tends to influence the proximal segment control and could cause clockwise rotation of the proximal segment (25). Finally, another source of inaccuracy in orthognathic surgery is the position of the mandibular condyle in the fossa during the scan of CBCT and actual surgical procedures (15). To reduce the possibility of an incorrect centric relation, temporomandibular joint placement must be identified during the preoperative evaluation (26). Another alternative would be planning to perform surgery in the mandible first sequence (26,27). Nevertheless, these relative errors could affect the results of this study.

A difference of less than 2 mm between the VSP and the actual postoperative hard tissue surfaces has been considered clinically acceptable (10,15,17,19,20). In the present study, the results rejected the null hypothesis that all values of mean deviations would be higher than 2 mm ( Table 3). It is important to highlight that 3D Error mean was 1,27 mm ( Table 2 and Fig. 4), and some additional information can be obtained taking into account the color maps (Fig. 3). A similar concept of the surface comparison performed by Tucker *et al.* which evaluated the accuracy of the VSP based on the surface distance differences between planning and actual outcomes on eleven different regions of the maxilla and mandible. Although this method accurately evaluated the effect of the surgery on the operating regions, maybe it would have been limited in the clinical application to answer the surgeon’s questions whether their planning had been fulfilling or not (6). Hernández-Alfaro and Guijarro-Martinez also used the ICP algorithm that provided a color scheme diagram to report the mean and standard deviations of the difference in distances between the surfaces, but this study only assessed the intermediate position of the two jaws while the intermediate splint and did not assess the comparison of the VSP to the surgical outcome.

In this study, the method partially satisfies the criteria suggested in the systematic review on a protocol for 3D accuracy evaluation of VSP in orthognathic surgery (6), which consisted in reducing the possibility of human error through voxel-based superimposition (using the cranial base as reference), semi automatically evaluating the results and validating the method and results by using intra and inter-evaluators reproducibility ( Table 2). However, the results showed only the anteroposterior direction and magnitude of deviations and did not fully describe the deviations in complex 3D, because it’s not presented translational or rotational based on the deviations axes (x, y, and z coordinates). As with some published in this area, the studies did not stratify the deviations by the three Cartesian frames of reference (x,y, and z) (11).

There are limitations regarding the ICP algorithm to evaluate accuracy between surface meshes of hard tissue 3D models (3,8,23). The major shortcoming of this approach lies in the fact that the distances were between the two nearest points of the two surfaces meshes (shortest deviations between vertices of the adjacent meshes), nor actual correspondence (28) or neither corresponding the same anatomical points (8,23). Another source of limitation may be correlated to erroneous data on the surface mesh (for example, streak artifacts or surface roughness) would have a marked effect on this measurement (8). The computation of artificial intelligence algorithms can have the challenge to overcome this limitation because relative errors can be caused by streak artifacts that were frequently present as the result of orthodontic appliances, which hampered an accurate automatic recognition of anatomical structures (29). Jabar *et al.* highlighted that the numerical values (mean distance and RMS) obtained are the Euclidean distances between points. The authors evidenced that a drawback of this current method should be taken into account when trying to assess 3D hard tissue changes (between pre and postoperative surface meshes of 3D models from the plastic skull) because their results have shown an underestimation of the magnitude of distances of simulated surgical movement by about 50 – 70%. Therefore, these limitations can affect the validity of the measurements (actual deviations).

In the present study, we could suggest that relative underestimation errors were reduced because the ICP algorithm was used on the similar surface meshes of 3D models matched on Geomagic Wrap software. In order to confirm this aforementioned hypothesis, another study of feasibility method must be carried out with different values of maximum and minimum deviation calibration on the same software. Hopefully, in future publications, new types of analysis will become available to understate the limitation and drawbacks, such as studies that facilitate automatic evaluation, softwares compatible with several VSP, and the recognition of methodologies for application in clinical trials for the assessment of the accuracy for virtually planned orthognathic surgery.

## Conclusions

This study showed 3D error mean (1,27 mm) within the standards of clinical success, lower than 2 mm. The ICP algorithm registration in Geomagic Wrap software provided a reproducible method of alignment between 3D models (surface meshes) and generated color maps to evaluate 3D qualitative congruence but did not answer all methodological parameters regarding the assessment of accuracy in orthognathic surgery.

## References

[1] Mazzoni S, Bianchi A, Schiariti G, Badiali G, Marchetti C (2015). Computer-Aided design and computer-aided manufacturing cutting guides and customized titanium plates are useful in upper maxilla waferless repositioning. J Oral Maxillofac Surg.

[2] Zinser MJ, Mischkowski RA, Sailer HF, Zöller JE (2012). Computer-assisted orthognathic surgery: feasibility study using multiple CAD/CAM surgical splints. Oral Surg Oral Med Oral Pathol Oral Radiol.

[3] Baan F, Liebreqts J, Xi T, Schreurs R, de Koning M, Bergé S (2017). A new tool for assessing the accuracy of bimaxillary surgery: the OrthoGnathicAnalyser. Plos One.

[4] Stokbro K, Aagaard E, Torkov P, Bell RB, Thygesen T (2014). Virtual planning in orthognathic surgery. Int J Oral and Maxillofac Surg.

[5] Haas Jr OL, Becker OE, Oliveira RB (2015). Computer-aided in orthognathic surgery – systematic review. Int J Oral and Maxillofac Surg.

[6] Gaber RM, Shareen E, Falter B, Araya S, Politis C, Swennen GRJ (2017). A systematic review to uncover a universal protocol for accuracy assessment of 3-dimensional virtually planned orthognathic surgery. J Oral Maxillofac Surg.

[7] Hernández-Alfaro F, Guijarro-Martínez R (2013). New protocol for three-dimensional surgical planning and CAD/CAM splint generation in orthognathic surgery: an in vitro and in vivo study. Int J Oral and Maxillofac Surg.

[8] Jabar N, Robinson W, Goto TK, Kambay BS (2015). The validity of using surface meshes for evaluation of three-dimensional maxillary surgical changes. Int J Oral Maxillofac Surg.

[9] Jayaratne YSN, Zwahlen RA, Lo J, Cheung LK (2010). Three-dimensional color maps: a novel tool assessing craniofacial changes. Surg Innov.

[10] Jayaratne YSN, McGrath C, Zwahlen RA (2012). How accurate are the fusion of cone-beam CT ad 3D Stereophotographic images?. Plos One.

[11] Mundluru T, Almukhtar A, Ju X, Ayoub A (2017). The accuracy of three-dimensional prediction of soft tissue changes following the surgical correction of facial asymmetry: an innovative concept. Int J Oral Maxillofac Surg.

[12] Weissheimer A, Menezes LM, Koerich L, Pham J, Cevidanes LHS (2015). Fast three-dimensional superimposition of cone beam computed tomography for orthopedics and orthognathic surgery evaluation. Int J Oral and Maxillofac Surg.

[13] Koerich L, Burns D, Weissheimer A, Claus JDP (2016). Three-dimensional maxillary and mandibular regional superimposition using cone beam computed tomography: a validation study. Int J Oral and Maxillofac Surg.

[14] Zhao YJ, Xiong YX, Wang Y (2017). Three-dimensional accuracy of facial scan for facial deformities in clinics: a new evaluation method for facial scanner accuracy. PLoS One.

[15] Ritto FG, Schmitt ARM, Pimentel T, Canellas JV, Medeiros PJ (2018). Comparison of the accuracy of maxillary position between conventional model surgery and virtual surgical planning. Int J Oral Maxillofac Surg.

[16] Proffit WR, Phillips C, Turvey TA (1987). Stability following superior repositioning of the maxilla by Le Fort I osteotomy. Am J Orthod Dentofacial Orthop.

[17] Marchetti C, Bianchi A, Bassi M, Gori R, Lamberti C, Sarti A (2006). A mathematical modeling and numerical simulation in maxillo-facial virtual surgery (VISU). J Craniofac Surg.

[18] Xia JJ, Gateno J, Techgraeber JF, Christensen AM, Lasky RE, Lemoine JJ (2007). Accuracy of the computer-aided surgical simulation (CASS) system in the treatment of patients with complex craniomaxillofacial deformity: a pilot study. J Oral Maxillofac Surg.

[19] Mazzoni S, Badiali G, Lancellotti L, Babbi L, Bianchi A, Marchetti C (2010). Simulation-guided navigation: a new approach to improve intraoperative three-dimensional reproducibility during orhtognathic surgery. J Craniofac Surg.

[20] Tucker S, Cevidanes LHS, Styner M, Kim H, Reyes M, Proffit W (2010). Comparison of actual surgical outcomes and 3-dimensional surgical simulations. J Oral Maxillofac Surg.

[21] De Riu G, Meloni SM, Baj A, Corda A, Soma D, Tullio A (2014). Computer-assisted orthognathic surgery for correction of facial asymmetry: results of a randomized controlled clinical trial. Br J Oral and Maxillofac Surg.

[22] Katkar RA, Kummet C, Dawson D (2013). Comparison of observer reliability of three-dimensional cephalometric landmark identification on subjects images from Galileos and i-CAT cone beam CT. Dentomaxillofac Radiol.

[23] Almukthar A, Khambay B, Ayoub A, Ju X, Al-Hiyali A, Macdonald J (2015). "Direct Dicom Slice Landmarking" A novel research technique to quantify skeletal changes in orthognathic surgery. PloS One.

[24] Nada RM, Maal TJ, Breuning KH, Bergé SJ, Mostafa YA, Kuijpers-Jagtman AM (2011). Accuracy and reproducibility of voxel-based superimposition of cone-beam computed tomography models on the anterior cranial base and the zygomatic arches. Plos One.

[25] Beukes J, Reyneke JP, Becker PJ (2012). Medial pterygoid muscle and stylomandibular ligament: the effects on postoperative stability. Int J Oral Maxillofac Surg.

[26] Perez D, Ellis 3rd E (2011). Sequencing bimaxillary surgery mandible first. Int J Oral Maxillofac Surg.

[27] Perez D, Ellis 3rd E (2016). Implications of sequencing in simultaneous maxillary and mandibular orthognathic surgery. Atlas Oral Maxillofac Surg Clin North Am.

[28] Cheung MY, Almukhtar A, Keeling A, Hsung TC, Ju X, McDonald J (2016). The accuracy of conformation of a generic surface mesh for the analysis of facial soft tissue changes. Plos One.

[29] Shahidi S, Bahrampour E, Soltanimehr E, Zamani A, Oshagh M, Moattari M (2014). The accuracy of a designed software for automated localization of craniofacial landmarks on CBCT images. BMC Med Imaging.

